# 

**Published:** 1858-08

**Authors:** 


APOLOGY AND BILLS.—The undersigned has delayed the issue of the present number, to carry out a determination to personally examine every man's account, and enclose the same in the Journal. It has cost much more time than we expected. The bills are made as they stand on our books. If any subscriber receives one he thinks erroneous, let him immediately inform us and we will be happy to make any corrections required. Let none infer from the delay of this number that we contemplate suspending the Journal. We shall *suspend* it to *those only* who fail to *pay* for it, as stated in the July number. All who *pay* will receive the Journal more promptly in future than any time heretofore.

N. S. DAVIS, *Proprietor.*

